# Q Fever: Current State of Knowledge and Perspectives of Research of a Neglected Zoonosis

**DOI:** 10.1155/2011/248418

**Published:** 2011-12-13

**Authors:** Sarah Rebecca Porter, Guy Czaplicki, Jacques Mainil, Raphaël Guattéo, Claude Saegerman

**Affiliations:** ^1^Research Unit in Epidemiology and Risk Analysis Applied to Veterinary Sciences (UREAR), Department of Infectious and Parasitic Diseases, Faculty of Veterinary Medicine, University of Liège, B42, Boulevard de Colonster 20, 4000 Liège, Belgium; ^2^Département de Sérologie, Association Régionale de Santé et d'Identification Animales, 4431 Loncin, Belgium; ^3^Laboratory of Bacteriology, Department of Infectious and Parasitic Diseases, Faculty of Veterinary Medicine, University of Liège, Sart-Tilman B43a, 4000 Liège, Belgium; ^4^UMR 1300 Bio-Agression, Epidémiologie et Analyse de Risque, INRA, 44307 Nantes, France

## Abstract

Q fever is an ubiquitous zoonosis caused by an resistant intracellular bacterium, *Coxiella burnetii*. In certain areas, Q fever can be a severe public health problem, and awareness of the disease must be promoted worldwide. Nevertheless, knowledge of *Coxiella burnetii* remains limited to this day. Its resistant (intracellular and environmental) and infectious properties have been poorly investigated. Further understanding of the interactions between the infected host and the bacteria is necessary. Domestic ruminants are considered as the main reservoir of bacteria. Infected animals shed highly infectious organisms in milk, feces, urine, vaginal mucus, and, very importantly, birth products. Inhalation is the main route of infection. Frequently asymptomatic in humans and animals, Q fever can cause acute or chronic infections. Financial consequences of infection can be dramatic at herd level. Vaccination with inactive whole-cell bacteria has been performed and proved effective in humans and animals. However, inactive whole-cell vaccines present several defects. Recombinant vaccines have been developed in experimental conditions and have great potential for the future. Q fever is a challenging disease for scientists as significant further investigations are necessary. Great research opportunities are available to reach a better understanding and thus a better prevention and control of the infection.

## 1. Introduction

Q fever was first described in 1935 in Queensland, Australia, during an outbreak of a febrile illness of unknown origin (Query fever) among abattoir workers [1]. It was subsequently classified as a “Category “B” critical biological agent” by the Centre for Diseases Control and Prevention and is considered a potential weapon for bioterrorism [2]. Q fever is a public health concern throughout the world [3]. While Q fever is an OIE notifiable disease, it remains poorly reported and its surveillance is frequently severely neglected. 

Q fever is a zoonotic bacterial disease. Domestic ruminants (cattle, sheep, and goats) are considered as the main reservoir for the pathogen which can infect a large variety of hosts, mammals (humans, ruminants, small rodents, dogs, and cats) and also birds, fish, reptiles, and arthropods [4–14]. It was reported to be a highly infectious disease in guinea pigs during experimental intraperitoneal infections [15, 16]. Both in animals and humans, however, Q fever infections remain poorly understood [17, 18] and their prevalence have been underestimated for many years [17].

## 2. Causal Agent

The causal agent of Q fever is *Coxiella burnetii*, an obligate intracellular Gram-negative bacterium of the *Legionellales* order, which was first observed as a rickettsia-like organism in the spleen and liver of mice inoculated with the urine of the abattoir workers [19–22]. Its predilected target cells are the macrophages located in body tissues (e.g., lymph nodes, spleen, lungs, and liver) and the monocytes circulating in the blood stream [23]. 

Two different antigenic forms of *Coxiella burnetii* can be distinguished [24]. The difference between phase I and phase II bacterial forms resides in the variation of the surface lipopolysaccharide (LPS) as classically described for enterobacteria [25]. Only phase I bacteria have a complete LPS on their surface and are virulent bacteria [26]. Phase I bacteria can be isolated from a naturally infected individual or from animals infected in a laboratory [27, 28]. On the other hand, phase II bacteria have an incomplete LPS due to a spontaneous genetic deletion of 25,992 bp [29] and are nonvirulent [28]. Phase II bacteria occur during serial passage in an immunologically incompetent host, such as cell cultures or fertilized eggs [27–29]. The deleted chromosomal region comprises a high number of genes that are predicted to function in LPS or lipooligosaccharide biosynthesis, as well as in general carbohydrate and sulfur metabolism [30]. However, in Australia, the study by Thompson et al. [29] and a later study by Denison et al. [31] on the genome of phase II human strains by polymerase chain reaction (PCR) reported the absence of truncated genes or of deletions. The Institute for Genomic Research has suggested that at least two other chromosomal regions are implicated in phase transition [29]. Antigenic variation of *Coxiella burnetii* is important for serological diagnosis and elaboration of vaccines. Indeed, serologically, anti-phase II antibodies (IgG and IgM) are found at high levels in acute Q fever, whereas anti-phase I antibodies (IgG and IgA) are found at high levels only during chronic infection [28]. 

Several genetic studies have been performed on *Coxiella burnetii*. The genome of the American Nine Mile strain was sequenced completely in 2003 [32]. The chromosome varies in size from 1.5 to 2.4·10^6^ base pairs and was highly variable among different *C. burnetii *strains. Occasionally, a 33 to 42 kb plasmid (depending on the plasmid considered) can be observed but its function remains to be determined [33]. Bacterial isolates can be identified by a probe to 16S ribosomal RNA (rRNA), which is highly conserved [34]. Genetic heterogeneity of *Coxiella burnetii* is limited with approximately 30 distinct variants [35]. According to experimental studies, bacterial strains vary in their pathogenic effect [36–39]. Masuzawa et al. [40] studied the macrophage infectivity potentiator gene (Cbmip of 654-base DNA) and the sensor-like protein gene (qrsA of 1227-base DNA) sequences between eleven strains. Their results demonstrated that Cbmip and qrsA sequences were highly conserved (>99%) and did not explain differences in pathogenicity [40]. Furthermore, three different plasmids have been identified in *Coxiella *variants [41]: QpH1, QpRS, and QpDG [42, 43]. Another plasmid (QpDV) has been isolated in a strain from a human case of endocarditis [44]. Plasmids differ by size and genomic sequence. However, several identical genomic sequences are present in all plasmids. In bacteria without plasmids, these sequences are found on the chromosome [41]. Generally, plasmids are of little interest for identification of microorganisms because they are not critical for survival and can infect a large variety of organisms [41]. However, *Coxiella burnetii* plasmids have proven to be useful because different strains contain different plasmids. In fact, QpH1, QpRS, and QpDV were present in different genotypes and were associated with difference in pathogenicity in the study by Frazier et al. [41]. Moreover, Savinelli and Mallavia [45] reported that, in human patients, the QpH1 and the QpRS plasmids (or plasmidless strains containing QpRS-related plasmid sequences) were associated with acute and chronic infection, respectively. However, later studies by genomic restriction fragment length polymorphism analysis, plasmid typing, or lipopolysaccharide analysis, on a larger number of strains did not confirm their results. Indeed, recent data shows that genetic variation has an apparent closer connection with the geographical source of the isolate than with clinical presentation [33, 46]. Moreover, host factors seem to be more important than genomic variation for development of acute or chronic infection [47, 48]. According to the recent report by the OIE [49], no specific genotype is associated with acute or chronic infection, with a particular clinical outcome, or with a specific host. 

## 3. Pathogenesis

The most important route of infection is inhalation of bacteria-contaminated dust, while the oral route is considered of secondary importance. Once inhaled or ingested, the extracellular form of *Coxiella burnetii* (or SCV after small-cell variant) attaches itself to a cell membrane and is internalized into the host cells. Phagolysosomes are formed after the fusion of phagosomes with cellular acidic lysosomes. The multiple intracellular phagolysosomes eventually fuse together leading to the formation of a large unique vacuole. *Coxiella burnetii* has adapted to the phagolysosomes of eukaryotic cells and is capable of multiplying in the acidic vacuoles [50]. In fact, acidity is necessary for its metabolism, including nutrients assimilation and synthesis of nucleic acids and amino acids [51]. Multiplication of *Coxiella burnetii* can be stopped by raising the phagolysosomal pH using lysosomotropic agents such as chloroquine [52, 53]. The mechanisms of *Coxiella burnetii* survival in phagolysosomes are still under study. Mo et al. [54] and Akporiaye and Baca [55] identified three proteins involved in intracellular survival: a superoxide dismutase, a catalase, and a macrophage infectivity potentiator (Cbmip). Redd and Thompson [56] found that secretion and export of Cbmip was triggered by an acid pH *in vitro*. Later, studies by Zamboni and Rabonovitch [57] and by Brennan et al. [58] demonstrated that growth of *Coxiella burnetii* was reduced by reactive oxygen intermediates (ROI) and reactive nitrogen intermediates. Hill and Samuel [59] analyzed *Coxiella burnetii*'s genome and identified 2 acid phosphatase enzymes. They demonstrated experimentally that both a recombinant acid phosphatase (rACP) enzyme and *Coxiella burnetii* extracts had a pH-dependent acid phosphatase activity. Moreover, rACP and bacterial extracts were capable of inhibiting ROI response by PMN despite their exogenous stimulation by a strong PMN stimulant. Inhibition of the assembly of the NADPH oxidase complex was found to be the mechanism involved [59]. *In vitro* studies on persistently infected cells with phase I and phase II bacteria reported a similar mitotic rate in infected and noninfected cells [23]. Moreover, the authors frequently observed asymmetric cellular divisions in infected cells and suggested that this phenomenon could allow maintenance of persistent infection [60]. 

The intracellular cycle of *Coxiella burnetii* leads to the formation of two development stages of the bacterium known as “small-cell variant” (SCV) and “large-cell variant” (LCV) [61–64]. SCV is the extracellular form of the bacterium. Typically rodshaped, SCVs are compact measuring from 0.2 to 0.5 *μ*m with an electron-dense core [61]. According to previous studies, SCVs are considered to be metabolically inactive and capable of resisting extreme conditions such as heat, desiccation, high or low pH, disinfectants, chemical products, osmotic pressure, and UV rays [19, 22, 62, 64, 65]. Their resistance in the environment (pseudospores) would enable the bacteria to survive for long periods of time in the absence of a suitable host. SCVs of *Coxiella burnetii* are reversible [17]. Indeed, once inhaled or ingested, the SCV attaches itself to a cell membrane and is internalized. After phagolysosomal fusion, the acidity of the newly formed vacuole induces activation of SCV metabolism and its development into LCV. During the morphogenesis from SCV to LCV, no increase in bacterial number is reported [66]. LCV is considered to be the metabolically active intracellular form of *Coxiella burnetii*. They are more pleomorphic than SCVs. Their cell wall is thinner and they have a more dispersed filamentous nucleoid region. They can exceed 1 *μ*m in length [61]. Intracellular growth is relatively slow with a doubling time of approximately 8 to 12 hours [24]. LCVs can differentiate into spore-like bacteria by binary asymmetrical division. The endogenous spore-like forms can undergo further development and metabolic changes until finally reaching the SCV form. Finally, cell lysis, or possibly exocytosis, releases the resistant bacteria into the extracellular media [67]. The physical and biological factors responsible for the sporulation-like process remain unknown. According to Rousset et al. [17], most natural infections by *Coxiella burnetii* are probably due to SCV or pseudospores present in the environment. Thus, decreasing the prevalence of Q fever infections requires a strict limitation of the environmental population of *Coxiella * pseudospores by using hygienic preventive measures [17]. Studies on the immune reaction in naturally or experimentally infected individuals have suggested that cellular immunity and the synthesis of IFN*γ* are essential for control of *Coxiella burnetii* infection [68–70]. Helbig et al. [69] demonstrated the predominant role of IFN*γ*, its level of production determining the outcome of infection. Indeed, IFN*γ* has been successfully tested to treat Q fever in patients not responding to antibiotic treatment [71, 72]. A study by Shannon et al. [70] reported that the development of protective antibody-mediated immunity *in vivo* was found to be independent of the cellular Fc receptors and of the complement [70]. The major part of vaccine-derived humoral response consists of IgG antibodies directed against proteins [73, 74]. Several studies report that natural humoral response to *Coxiella burnetii* is directed against both protein and glycolipid fractions [75–78]. Chen et al. [79] identified 8 new CD4+ T-cell epitopes. However, all the CD4+ T cell epitopes did not lead to B-cell stimulation and specific antibody production. Koster et al. [80] reported that, in chronic infections, peripheral blood lymphocytes do not proliferate when exposed to *Coxiella burnetii* antigens despite proliferating when exposed to other antigens or mitogens. This was not observed in acute infection [81]. In addition, Shannon et al. [82] observed that *Coxiella burnetii* phase I cells appeared almost invisible to dendritic cells. 

Hormonal changes during pregnancy cause immunomodulation in the female body causing reactivation of the organism. This immunomodulation has been advanced as an explanation for the increased multiplication of the organism in the placenta [83].

Further research is more than necessary to fully understand the complex processes developed by *Coxiella burnetii* to enter and infect a specific host cell, to resist in the intracellular and extracellular environment, and its ability to cause illness. Moreover, a better understanding of the immune system reaction to infection would give an insight into the processes developed by the bacterium and by the host that determine the final outcome of disease (asymptomatic, acute, or chronic).

## 4. Epidemiological and Clinical Aspects

### 4.1. Routes of Infection

Inhalation is the most common route of infection in both animals and human [84–86]. Under experimental conditions, inhalation of a single *C. burnetii* can produce infection and clinical disease in humans [87]. However, similar studies have not been done in animals [88]. As mentioned above, domestic animals are considered the main reservoir for the pathogen [89–91]. Infected animals contaminate the environment by shedding *Coxiella burnetii* in milk, feces, urine, saliva [13, 92], and very importantly in vaginal secretions, placenta, amniotic fluids, and other products of conception [13, 92–99]. *Coxiella burnetii* also spreads by wind causing infections at a distance from the initial source of bacteria [93, 96, 100, 101]. 

In domestic ruminants, milk is the most frequent route of pathogen shedding [97]. Currently, controversy remains concerning the possibility of infection by oral route [102]. Results of previous studies on the subject are considered inconclusive [7, 103–106]. OIE advises not to drink raw milk originating from infected farms [49]. Further research is required to clarify the probability of infection by oral route. If infection by oral route is proven to be efficient, the sufficient number of pathogens capable of causing Q fever should be determined [17]. 

Human-to-human transmission does not usually occur [49, 107] although it has been described following contact with parturient women [108, 109]. In addition, cases of sexual transmission of Q fever have been reported [110–112]. Currently, risk of transmission through blood transfusion is considered negligible [113, 114]. Transplacental transmission, intradermal inoculation, and postmortem examinations have been associated with sporadic cases of Q fever [115–118].

### 4.2. Q Fever in Domestic Animals and Wildlife

#### 4.2.1. Cattle

Q fever is widespread in livestock, and its seroprevalence is thought to have increased in recent years [33]. Often neglected in the differential diagnosis, Q fever can persist in a herd causing great financial losses on the long term [94]. 

In ruminants, well-known manifestations of Q fever are abortion, stillbirth, premature delivery, and delivery of weak offspring [3]. However, these dramatic clinical manifestations are generally only expressed in sheep and goats. In cattle, Q fever is frequently asymptomatic. Clinically infected cows develop infertility, metritis, and mastitis [94]. In addition, *C. burnetii* was found to be significantly associated with placentitis [119, 120]. Placental necrosis and fetal bronchopneumonia were also significantly associated with the presence of *Coxiella burnetii* in the trophoblasts [119]. Unlike humans and experimentally infected cows [121], naturally infected ruminants rarely present respiratory or cardiac signs. Beaudeau et al. [122] and Guatteo et al. [92] performed respective studies on shedding of *Coxiella burnetii* by infected cows. In the study by Guatteo et al. [92], the apparent proportion of shedders in clinically infected dairy herds among the cows sampled was 45.5%. Milk was the most frequent positive sample for the bacterium compared to feces and vaginal mucus samples. The percentage of positive samples of each type was 24.4, 20.7, and 19%, respectively. 65.4% of sampled cows excreted by one shedding route only, whereas 6.4% shed bacteria in the vaginal mucus, feces, and milk simultaneously. A combined shedding in vaginal mucus and in feces and in vaginal mucus and milk were observed in 14.6% and 10% of cases, respectively [92]. The study by Beaudeau et al. [122] reported, within endemic infected dairy herds, that 85% of their infected cows excreted by one shedding route only. In their study, only 2% of the infected cows shed bacteria in the vaginal mucus, feces and milk simultaneously. When combined shedding occurred, the combination of shedding in vaginal mucus and milk was the most frequently observed. The results of these two French studies are very similar and the slight differences observed could be due to differences in the sampled population of cattle. Furthermore, different areas can have variable prevalence of Q fever and not only is shedding in milk intermittent and its outset not associated with parturition, but also it differs from one herd to another despite species being identical [97]. With milk being such a major shedding route, bulk tank milk (BTM) samples are useful for investigation of the sanitary grade of bovine [123], ovine [124], and caprine herds [125]. Indeed, polymerase chain reaction (PCR) and enzyme-linked immunosorbent assay (ELISA) performed on BTM samples, in addition to the analysis of serum samples of at least 10% of the animals in the herd, give rapid, economical, and valuable information on the herd's status [97, 126]. In cows' milk, Marrie [127] identified *Coxiella burnetii* for up to 32-month postpartum. Vaginal and fecal bacterial discharges seem to have a major impact on environmental contamination as a result of practices at kidding and effluent management [88, 97]. Indeed, the incidence of Q fever has been observed to increase significantly during the lambing period (winter and early spring) [9]. A seasonal correlation with spread of goat manure was reported in The Netherlands during the outbreaks of 2007-2008 [114]. 

Epidemiological data has shown that cows are more frequently chronically infected than sheep with persistent shedding of bacteria as a result [89]. The sites of chronic *Coxiella burnetii* infection are the uterus and the mammary glands of females [20, 24]. Moreover, heifers are less frequently infected than older animals even in infected herds [128, 129] making those a preferential group for effective vaccination programs.

#### 4.2.2. Goats

Numerous studies have suggested that epizootics of Q fever in goats are related to outbreaks in humans [99, 114, 130, 131]. Indeed, in many countries, goats are the most common source of human infection due to their extensive raising and close contact with humans [98]. 

Q fever in goats can induce pneumonia, abortions, stillbirth, and delivery of weak kids, the latter two clinical signs being the most frequently observed [98, 99, 132]. Abortions occur principally nearing the end of the pregnancy [99]. The frequency of occurrence of Q fever abortions in goats is more important than in sheep with up to 90% of females being affected [98]. Moreover, a study by Berri et al. [98] reported that pregnancies subsequent to abortion may not be carried to term. Similar to cattle, pregnant animals are more susceptible to infection than nonpregnant animals [98]. These animals also frequently develop chronic infections with persistence of the bacteria in the uterus and mammary glands [133]. Shedding of *Coxiella burnetii* by infected goats is discontinuous [98, 99, 134–136]. In naturally infected goats, shedding seemed to be limited to the kidding season following infection [137]. However, an experimental study performed by Berri et al. [98] reported that infected animals can shed *Coxiella* during two successive kidding seasons in vaginal mucus and milk. Moreover, shedding in milk can be maintained for a long period [98, 136]. Milk has been considered the major route of bacteria excretion for this species by several authors [97]. 

De Cremoux et al. [138] in their recent study on bacterial shedding in vaginal mucus observed persistence of shedding at the subsequent kidding season following a Q fever outbreak despite a reduced number of clinical signs. Similar to cattle, susceptible or naïve animals were more common among kids than adults [139].

#### 4.2.3. Sheep

Sheep have a predisposition for abortion similar to goats [13, 98]. However, Q fever in sheep seldom causes chronic infections [13, 17, 132]. Infected sheep, like goats, shed *Coxiella burnetii* in vaginal secretions, urine and feces, and to a lesser extent in milk [97]. In naturally infected sheep, the bacterium has been isolated in vaginal discharges long after abortion [140] and can be shed at subsequent pregnancies [141]. In the study by Rodokalis et al. [97], no ewe was found to shed bacteria constantly in milk. Some sheep have been found to shed *Coxiella burnetii* during 11 to 18 days postpartum in feces [127]. Most ewes shed bacteria by at least two routes, mainly in feces and vaginal mucus [97]. Rodokalis et al. [97] reported a flock where no ewe ever shed by a single route (no exclusive shedding in milk or feces or vaginal mucus). 

In certain areas (e.g., Basque country), sheep are an important source of human infections. Garcia-Pérez et al. [124] studied the presence of *Coxiella burnetii* DNA in BTM and its association with seroprevalence. The authors reported a lower seroprevalence in lambs and yearlings compared to older ewes. Herds with a history of abortion had a higher seroprevalence than other herds, but the difference observed was not significant. Flocks with a level of seropositivity superior to 30% were more frequently positive on BTM-PCR analysis. However, flocks with a low number of seropositive animals but with a positive result at BTM-PCR analysis were also observed in this study [124]. 

In France, two cases of human Q fever were associated with the use of ovine manure as garden fertilizer. The flock of sheep that had provided the manure did not present any clinical signs, despite the presence of seropositive animals and excretion of bacteria in feces [101]. The Q fever outbreak that occurred in Bulgaria in 2004 was also associated with infected sheep and goats [142]. 

Astobiza et al. [143] studied the effect of vaccination with a phase I inactivated vaccine (Coxevac). After vaccinating 50% of replacement lambs and 75% of ewes in two naturally infected sheep flocks, the number of abortions, shedders and the general bacterial load were significantly diminished. However, this measure did not prevent seroconversion of nonvaccinated lambs and identification of *Coxiella burnetii *in aerosols obtained in the sheep's environment. This confirms that vaccination must thus be associated with other preventive and control measures in infected environments.

#### 4.2.4. Pigs

Natural susceptibility has been demonstrated by the presence of antibodies to *Coxiella burnetii* in their serum [144]. However, the role played by pigs in the epidemiology of Q fever remains unknown [13].

#### 4.2.5. Cats and Dogs

Dogs and cats can be infected by Q fever and have been associated with human infections in rural and urban areas [145–155]. In 1952, Gillepsie and Baker [156] performed successful feline experimental infections by subcutaneous inoculation, feeding infected yolk sacs, and by contact with infected cats. Despite the presence of pathogen in blood, urine, and a serological response, clinical signs were not observed in all infected cats. In felines, Q fever is, seemingly, frequently asymptomatic and remains undiagnosed. However, infected cats excrete bacteria in the environment and become a potential source of human infections. In Japan, cats are widely considered to be one of the most important reservoirs of *Coxiella burnetii* [157]. The study by Komiya et al. [157] found that seroprevalence was significantly higher in stray cats than in domestic cats. Thus, the feline environment seems to influence the probability of *Coxiella* infection [157].

The potential importance of dogs for the transmission of Q fever to humans is rarely mentioned. Dogs are however as close, if not closer, to humans as cats. Dogs can potentially be infected by inhalation, tick bites, consumption of placentas, or milk from infected ruminants. Buhariwalla et al. [148] reported three human cases of Q fever associated with an infected parturient dog. The puppies all died within 24 hours of birth. Indeed, Q fever in parturient dogs has previously been associated with early death of the pups [145]. Similar to cats, a previous study on dogs in California reported a higher prevalence of infection in stray than in domestic dogs [158]. 

Currently, the effect of infection and its clinical presentation remain poorly investigated in felines and canines.

#### 4.2.6. Horses

In previous studies, horses have been found to be seropositive toward Q fever [158]. Indeed, the study by Willeberg et al. [158] reported a seroprevalence of 26% (31/121) in horses in California. To our knowledge, no human case of Q fever has been associated with equids. Moreover, Q fever is not investigated routinely in cases of infertility or obstetric complications in this species.

#### 4.2.7. Wild Animals

At present, wildlife is considered of minor importance for Q fever epidemiology. Many wild mammals and birds have been found to be hosts to the infectious organism [13, 159–163]. A few cases of transmission of *Coxiella burnetii* from wild animals to humans have been reported but merit further experimental research [11, 13].

#### 4.2.8. Ticks and Other Potential Vectors

Among ectoparasites, ticks are considered to be the natural primary reservoir of *Coxiella burnetii*. Over 40 tick species are naturally infected [39, 145, 163–171]. Experimental infections have been obtained in guinea pigs with *Ixodes holocyclus*, *Haemaphysalis bispinosa*, *Rhipicephalus sanguineus,* and *Dermacentor andersoni*. In Europe, *Ixodes ricinus* is the most common tick and *Rhipicephalus sanguineus* is frequent on dogs [102, 168, 170]. Thus, both these species of ticks could be (or become) reservoirs of *Coxiella burnetii* in Europe.

Ticks excrete bacteria in saliva and feces. After multiplying in the cells of the midgut and stomach of an infected tick, extremely infectious bacteria are deposited onto the animal skin during fecal excretion. The feces are extremely rich in bacteria and may reach a concentration of 10^12^ organisms per gram [20, 89]. Furthermore, transovarial transmission is suspected as bacteria have been isolated in the ovaries of infected ticks [20]. However, despite all these factors, ticks are not thought to contribute to the maintenance of coxiellosis in endemic areas [13, 17]. Moreover, in the study by Astobiza et al. [162], none of the ticks analyzed were positive when tested by PCR despite the area being endemic for Q fever. Similarly, in two recent studies in endemic areas, a low percentage of *Dermacentor spp.* [172] and *Ixodes ricinus* [173] were found to be infected. Nevertheless, ticks are suspected of having a significant role in the transmission of *Coxiella burnetii* among wild vertebrates, especially rodents, lagomorphs, and wild birds [20, 89, 174]. In addition, Hirai and To [13] hypothesized that they could play a role for transmission of *Coxiella* from infected wild animals to domestic naive animals. Human infections through tick bites are rarely reported. Sporadic isolations of *Coxiella burnetii* in chiggers, lice, and flies have been reported [164, 175, 176].

### 4.3. Q Fever in Humans

The main characteristic of Q fever in humans is its clinical polymorphism [1, 15, 20]. Q fever is therefore considerably underdiagnosed and underreported [177]. After an incubation period of 1 to 3 weeks [33, 107], Q fever can cause either an acute or a chronic disease. Size of the inoculum, geographical area, route, and time of infection, as well as host factors influence the duration of the incubation period [15, 178, 179] and may contribute to the clinical expression of acute or chronic infection [20, 127]. 

#### 4.3.1. Acute Q Fever

In the acute form, infections can be totally asymptomatic in 50 to 60% of cases or cause a self-limiting illness associated with fever, fatigue, headache, and myalgia (influenza-like syndrome). When clinically expressed, acute fever is frequently accompanied by atypical pneumonia and/or hepatitis. Pneumonia is an important manifestation of Q fever in humans [1, 15, 20]. It is uncommon in Australia and some parts of Russia, whereas in North America and Europe it is the major manifestation of infection [93, 96]. Pneumonia is typically mild, but progression to acute distress syndrome can occur [107, 180, 181]. Endocarditis can exceptionally be associated with acute infection in 0.76% of cases [182, 183]. In pregnant woman, Q fever can lead to spontaneous abortion, intrauterine fetal death (IUFD), premature delivery, or intrauterine growth retardation. Transplacental infection of the fetus in utero has also been reported [109, 184, 185]. Moreover, Carcopino et al. [186] found that Q fever was significantly associated with oligoamnios, which is a recognized cause of neonatal morbidity and mortality [187, 188]. One case of endocarditis has been reported in a pregnant women with a bioprosthetic aortic valve and led to maternofetal death at 27-week gestation [186]. Infection during the first trimester of the pregnancy is particularly associated with a negative outcome [185, 186]. After infection, breast feeding is of course contraindicated [185]. 

Mortality rate of acute Q fever is estimated at 1 to 2% [107, 114, 189]. Myocarditis, occurring in less than 1% of cases, is the first cause of death [190]. As yet, myocarditis has not been reported in pregnant women [191].

#### 4.3.2. Chronic Q Fever

Chronic Q fever consists in the persistence of infection for more than 6 months. Chronic Q fever concerns 5% of infected individuals [192]. Most commonly, endocarditis is observed in 60–70% of cases of chronic infections [193], but chronic hepatitis, osteomyelitis, septic arthritis, interstitial lung disease, chronic fatigue syndrome [194], or infection of aneurysm and vascular grafts can also occur [1, 15, 20]. Q fever-associated endocarditis has been estimated to be responsible for 3 to 5% of all cases of human endocarditis [182, 195]. Individuals with underlying valvulopathy or other cardiovascular abnormalities are predisposed to the development of endocarditis [182, 183]. Over recent years, the occurrence of rare clinical manifestations such as osteomyelitis, optic neuritis, pericarditis, lymphadenopathy, and Guillain-Barre has significantly increased. Meningitis, encephalitis, polyradiculoneuritis, peripheral neuropathy, cranial nerve deficiency, optic nevritis, and paralysis of the oculomotor nerve have been reported in 3.5% of infected patients. The main clinical signs in neurological cases are headaches, behavioral problems, cognitive deficiency, or confusion. Convulsions and even epileptic fits and aphasia are possible [196–198]. In pregnant women, chronic Q fever can lead to spontaneous abortions in future pregnancies [109, 117, 199]. The prognosis of chronic infections is less favorable than for acute infections [107]. Antibiotic treatment is less effective, and the disease is usually long with mortality rates that can reach more than 50% [107].

#### 4.3.3. Q Fever in Children

In children, Q fever is thought to be rare. This could be explained by frequent asymptomatic or nonspecific presentations of infection leading to undiagnosed cases of *Coxiella* infection. Cases of hepatitis and pneumonia have however been reported in children and can be fatal [93, 200]. In Northern Ireland, McCaughey et al. [201] reported a seropositivity rate inferior to 10% in children. The seropositivity rate increased markedly during the late teenage years and especially in young adults [201]. Increased exposure with age is a plausible hypothesis to explain this phenomenon.

#### 4.3.4. Postillness Followup

The study by Limonard et al. [202] on followup of patients after the Q fever outbreak in The Netherlands reported fatigue in 52% of patients 6 months after illness and in 26% one year after illness. They observed very high level of anti-phase I and anti-phase II antibodies up to 3 months after the onset of disease, then a gradual decrease in the following 9 months. Post-Q fever chronic fatigue syndrome has also been reported [114, 194, 203]. This syndrome has been attributed to dysregulation of cytokine production, induced by persistent antigens including LPS and proteins, rather than persistent latent *Coxiella *[203].

#### 4.3.5. Predispositions

Recent experimental data indicates that host factors rather than specific genetic bacterial determinants are the main factors influencing the clinical course of *Coxiella burnetii* infection [14, 47, 195, 204]. 

In humans, Q fever is mainly considered an occupational hazard. A study by Whitney et al. [205] on 508 American veterinarians reported a prevalence rate (22.2%) far superior to the prevalence rate in the general adult US population. Veterinarians from a mixed or large animal practice were significantly more likely to be seropositive than veterinarians from a small animal practice. Furthermore, living on a farm, in the past or present, increased the probability of being seropositive for coxiellosis. Absence of protective clothing or mask, occupational risks (accidental cuts and needle sticks), and routine contact with water were also demonstrated as significant risk factors for infection [205]. McQuiston and Childs [206] reported a seroprevalence of 7.8% among American veterinarians, farmers, slaughterhouse workers, and tannery workers. Furthermore, a Northern Irish study reported a seropositivity rate significantly higher among farmers than in the general population (*P* < 0.001) [201]. Seropositivity was found to be slightly, but significantly, higher in males than in females (*P* = 0.023). In The Netherlands, during the Q fever outbreak in May 2010, people living within 2 km of the infected dairy goat farm had a much higher risk for Q fever than those living more than 5 km away [207].

On the contrary to countries where cats are the main reservoir of *Coxiella burnetii* where no sex predisposition is reported, men between 30 and 70 years of age are the most frequently affected by clinical Q fever in countries where cattle are considered the main reservoir [208–214]. The study by Raoult et al. [204] reported that being a male and over the age of 15 increased the risk of symptomatic disease. In Australia and France, males are 5-fold and 2.5-fold more likely than females to develop disease, respectively [195]. Although the precise reason for this difference remains unknown, it has been suggested that sex hormones play a role [195, 204]. Indeed, Leone et al. [215] reported a protective role of 17beta-oestradiol towards Q fever infection rendering females more resistant. Surprisingly, in a comparative study on clinical expression and outcome of Q fever after the outbreak in Chamonix Valley in France, pregnant women were found to be significantly less frequently symptomatic than other women and other patients (9.1% of pregnant women were symptomatic versus 90.6% of men, 75% women, and 33.3% children) [216]. Endocarditis is more frequently observed in men over 40 years of age [217]. Immunocompromised hosts are at risk of severe disease and of development of chronic infection. Furthermore, people with heart valve lesions, vascular abnormalities, liver cirrhosis, and cancer are more susceptible to developing chronic Q fever [183, 218]. In the study by Carcopino et al. [186], 52.8% of the pregnant women with acute Q fever developed chronic serologic profiles.

#### 4.3.6. Treatment

In nonpregnant women and other patients with acute Q fever, treatment consists in a daily dose of 200 mg doxycycline for 14 to 21 days. Hydroxychloroquine can be associated with doxycycline. Hydroxychloroquine increases the pH of the phagolysosomes, and its association with doxycycline has a bactericidal effect [219, 220]. Rifampin, erythromycin, clarithromycin, and roxithromycin can also be used as an alternative treatment [221, 222]. Fluoroquinolones are recommended in cases of meningoencephalitis as their penetration into the central nervous system is better compared to doxycycline. As mentioned previously, treatment with IFN*γ* has proven effective in certain cases [71, 72]. 

Treatment of chronically infected patients consists in a daily dose of 200 mg of doxycycline associated with 600 mg of hydroxychloroquine for duration of 18 to 36 months. The dosage of hydroxychloroquine is adjusted three months after initiation of treatment to obtain a plasmatic level of 1 ± 0.2 mg/L [219]. During the treatment, an ophthalmologic followup is recommended every 6 months to detect accumulation of hydroxychloroquine in the retina. Rolain et al. [223] advised a plasmatic concentration of 5 mg/L of doxycycline for effective treatment. Minimal inhibition concentration (MIC) for doxycycline against *Coxiella burnetii* varies from 1 to 4 *μ*g/mL [220]. Bacterial resistance to doxycycline was observed by Rolain et al. [224, 225] in a patient with endocarditis. Thus, serological testing on a regular basis to evaluate response to treatment is advised. It is considered that an anti-phase I IgG titer inferior to 200 is predictive of a clinical cure [226]. A decrease of 2 dilutions in antibody titers in one year at the minimum signifies successful evolution [220].

In infected pregnant women, administration of long-term cotrimoxazole (320 mg trimethoprim and 1600 mg sulfamethoxazole for at least 5 weeks), a bacteriostatic antibiotic, is advised [186]. After delivery, a daily treatment with 200 mg of doxycycline associated with 600 mg of hydroxychloroquine for 1 year minimum is advised for chronically infected women. The study by Carcopino et al. [186] reported that long-term cotrimoxazole therapy significantly reduced the number of obstetric complications, the frequency of placentitis, and the development of a chronic serological profile. In their study, no IUFD was observed in treated women (0% versus 27% of IUFD in nontreated women). The complications reported were intrauterine growth retardation and premature delivery. No spontaneous abortions and no oligoamnios occurred [186].

#### 4.3.7. Animal Models for Human Q Fever

Animal models for human infection are commonly laboratory mice and guinea pig models. Guinea pigs are regarded as a model of acute Q fever in humans [227]. On the contrary to guinea pigs, mice develop a chronic infection, although endocarditis does not occur in normal adult mice. They remain chronically infected for months and excrete bacteria in feces and urine. Their susceptibility to infection varies in function of the mouse strain [228]. Extrapolations of observations on experimentally infected laboratory rodents to naturally infected humans could prove erroneous in some aspects, and caution is recommended. Gonder et al. [229] and Waag et al. [230] reported a successful infection by aerosol route in cynomolgus and rhesus macaques. After infection, the macaques developed fever and pneumonia after 4 to 7 days. Their clinical expression of Q fever is thus unsurprisingly the closest to human clinical expression.

#### 4.3.8. Outbreak in The Netherlands

In 2009, infected goats were at the origin of 2,361 human cases of Q fever (including six deaths) diagnosed in The Netherlands [231]. Most cases were diagnosed in the North Brabant Province. A recent increase in high intensity dairy goat farming has lead to development of very large densely populated farms: from 100,000 goats in 2000 to 230,000 goats on approximately 350 farms in 2009 [232]. Transport of animals, manure spreading, and wind have been reported as influential factors for human infections. In a study by Karagiannis et al. [233] on the outbreak in 2007 in The Netherlands, living east of the area in which a positive goat farm, cattle, or small ruminants were situated, smoking and contact with agriculture products were found to be associated with recent infection. Scientists suspected the emergence of a more virulent subtype of *Coxiella burnetii* than the initial subtype of bacterium identified [131, 231]. Genomic studies reported that multiple genotypes were involved in the Q fever outbreak. However, an identical subtype was identified in a dairy goat herd suffering from multiple abortions and in several human patients [131]. This particular subtype could thus have a survival and propagation advantage compared to other bacterial subtypes [131, 231]. Roest et al. [234] performed multiple locus variable tandem repeat analyses on a large number of Q fever positive samples originating from domestic ruminants and associated with the outbreak. Their study found that one genotype predominated on all dairy goat farms in the southern part of The Netherlands. Indeed, on 12 out of 14 dairy goat farms, this genotype was found in 91% of samples, varying from 33% to 100%. Nine other genotypes occurred once, each representing only 0.8% of all found genotypes on the farms. The predominant genotype was found on 11 farms in the southern Netherlands and on a farm in the eastern part of the country. This suggests a clonal spread of *Coxiella burnetii* with this predominant genotype. However, another study by Huijmans et al. [235] found five distinct genotypes (3 in humans and 4 in livestock) implicated in the outbreak and concluded that environmental factors such as animal and human density and climate, rather than one particularly virulent strain favored the Dutch outbreak and its spread. Control measures were put in place following the outbreak. Vaccination of goats with Coxevac vaccine on voluntary grounds began in 2008. Vaccination was exclusively performed in areas of the human outbreak. In 2009, vaccination of all dairy sheep and goats on farms with more than 50 animals became mandatory, and area of vaccination was enlarged (North-Brabant province and parts of adjacent provinces). Moreover, BTM testing was performed every two weeks. A farm was considered infected when 2 consecutive BTM samples were positive by PCR. Movement and breeding were banned for dairy goats and sheep on infected farms [236]. Moreover, culling of all pregnant goats and sheep on these farms was also in application until May 2010.

## 5. Diagnosis

### 5.1. Direct Diagnosis

Direct diagnostic methods identify the presence of the bacterium or of one of its components.

#### 5.1.1. Coloration and Direct Visualization

Direct visualization of *Coxiella burnetii* on smears or frozen tissue is a method of Q fever diagnosis. Smears are taken from the placenta of aborted ruminants, from the fetus stomach content, or from other body tissues. *Coxiella burnetii* does not stain reliably with Gram stain, and Gimenez stain is preferentially used [237]. A Stamp-Macchiavello coloration, commonly called Macc staining, or routine Giemsa stain can also be performed. The specificity and sensitivity of direct visualization by bacterioscopic examination is poor, due to possible confusion with other pathogens such as *Brucella spp*., *Chlamydophila spp., *or *Chlamydia spp*. [92].

#### 5.1.2. Immunohistochemistry (IHC)

IHC has been performed for the diagnosis of chronic cases of Q fever. It can be utilized for detection of *Coxiella burnetii* in tissues fixed in paraffin or acetone smears [238]. Dilbeck and McElwain [239] developed an avidin-biotin-peroxidase complex IHC staining method for diagnosis and routine screening of ovine and caprine placental tissues after abortion. This technique is rapid and does not necessitate live bacteria or fresh tissues for diagnosis. Furthermore, it renders retrospective studies on stored samples possible. However, IHC is not useful for large-scale epidemiological studies.

#### 5.1.3. Bacterial Culture

*In vitro* cell culture of the bacteria is the gold standard for diagnosis of bacterial infections. *Coxiella burnetii* can be cultured efficiently in the yolk sac of chicken embryos and also on diverse cellular specimens, such as human embryonic lung fibroblasts, mosquito cells, green monkey kidney (Vero) cells, L cells, tick tissue cultures, and so forth [24]. However, technically, culture of *Coxiella burnetii* remains a difficult process and sensitivity of this diagnostic method is low. Recently, *Coxiella burnetii* can be grown outside a host cell in a cell-free laboratory medium [240]. The latter cell-free medium has a composition that strictly corresponds to the organism's metabolic requirements in the phagolysosome (*in vivo Coxiella* is strictly intracellular). This finding is revolutionary and will permit further studies on *Coxiella burnetii*. Another practical limitation to bacterial isolation is that it requires a Biosafety Laboratory 3 because of its high infectivity. As a result, culture is rarely performed, especially in veterinary medicine [192]. Moreover, epidemiological studies based on bacterial isolation are not practical.

#### 5.1.4. Polymerase Chain Reaction (PCR)

PCR offers substantial benefits for the identification of *Coxiella burnetii* compared to other laboratory techniques, especially in the early clinical stages of the illness [241, 242]. It has been successful in detecting *Coxiella burnetii* DNA in various samples, including cell cultures, biopsy samples, blood, arthropods, and serum samples [193, 243]. Its sensitivity and specificity are high. However, the usefulness of conventional PCR is limited by its inability to quantify the bacteria present. The development of real-time quantitative PCR (RTq PCR) not only renders PCR a rapid diagnostic tool but also provides quantifiable information. RTq PCR can be automated and thus can be used in large-scale studies. The qualities of PCR make it very useful for early diagnosis of infection during the period when antibodies are not yet present [242]. Several primers are available for diagnosis [241, 244–246]. A primer originating from the frequently repeated DNA sequence IS*1111* (7 to 120 copies per genome) is commonly used and has proven to allow very sensitive testing [241, 246]. In a study by Schneeberger et al. [246], *Coxiella burnetii* DNA was detected in 98% of seronegative acute Q fever patients and in 90% of anti-phase 2 IgM seropositive patients. PCR became progressively negative as the serological response developed [246]. Hou et al. [247] noticed a significant association between the absence of IgM antibodies and a positive PCR result. Negative serology was most frequent in Q fever cases where sampling had been performed within the two weeks of illness. In such cases, the authors recommend routine PCR testing as well as serology. When PCR was included in the diagnostic procedure primarily based on serology, a marked increase of sensitivity was observed (78% versus 29% when serology was used as unique diagnostic method) [247]. PCR and RTq PCR are considered methods of choice for the DNA detection in diverse samples [122]. However, PCR and RTq PCR are incapable of distinguishing bacterial DNA from live and/or dead bacteria [248]. The advantage is that sampled tissues can be frozen, put into formalin, or fixed with paraffin [242]. The disadvantage is that interpretation of positive results in fresh samples can be difficult [107, 249]. PCR kits are gradually becoming available [250]. Analysis of samples by PCR should be performed on sampling day to minimize the risk of obtaining false-negative results. Indeed, the study by Guatteo et al. [90] reported that only two-thirds of milk and one-half of vaginal mucus samples that were initially positive on the day of sampling remained positive after storage at −20°C during three days. Samples found to be persistently positive had a significantly higher estimated initial bacterial titre than those which became negative after storage.

### 5.2. Indirect Diagnosis

Indirect diagnostic methods identify specific humoral or cellular immunity in response to *Coxiella burnetii* infection. The diagnostic methods available in human and veterinary medicine differ, and little information is available concerning epidemiological sensitivity and specificity of diagnostic methods, especially in ruminants (Table 1).

#### 5.2.1. Complement Fixation Test (CFT)

In veterinary medicine, CFT was the method of reference for serological diagnosis according to OIE [102, 255, 256]. CFT usually utilizes phase 2 antigens only [27, 102]. It is capable of detecting approximately 65% of infected subjects during the second week after initial clinical signs and 90% during the fourth week [49]. CFT is more laborious, less specific, and less sensitive than indirect immunofluorescence assay (IFA) [192] or ELISA (described hereafter) [192]. A study by Rousset et al. [257] on goats originating from different herds reported that CFT results obtained on sera of aborting goats and of nonaborting goats were not significantly different and confirmed the lack of sensitivity of CFT compared to ELISA. CFT, on the contrary to ELISA, is incapable of detecting all IgG subclasses. In ruminants, only IgG1 fixes the complement and can thus be detected by CFT. Moreover, IgG2, IgM and anticomplement substances potentially present in sera are capable of interfering with fixation of IgG1 to the complement lowering the titer of IgG1 detected by CFT [99]. Rousset et al. [257] advised not to use CFT for serological animal screening because of its low sensitivity.

#### 5.2.2. Enzyme-Linked Immunosorbent Assay (ELISA)

Another method of diagnosis for human and animal cases is ELISA. This method is more sensitive and easier to perform, and to standardize than CFT [192]. Moreover, a strong association was reported between strongly positive ELISA results and the occurrence of abortion in goats [257]. As mentioned above, the higher sensitivity of ELISA compared to CFT could be due to the detection of the IgG_2_ subclass of antibodies, which are incapable of binding to the guinea pig complement [27]. Commercially available human ELISA kits are frequently coated by phase 1 and phase 2 antigens. The antigens present are of two possible origins: antigens of the American Nine Mile strain of *Coxiella burnetii* isolated from an endogenous tick or antigens of a strain originating from infected European domestic ruminants. ELISA kits coated by the latter antigens are more sensitive and are advised for serological diagnosis [258]. In France, the ELISAs available for veterinary diagnostic purposes do not differentiate anti-phase 1 and anti-phase 2 antibodies but detects total antibodies [102]. ELISA tests can be automated which facilitates large-scale studies [257]. 

In the study by Guattéo et al. [126], persistent shedder cows had mainly a persistently highly seropositive status (20 out of 27 and 17 out of 23 for weekly and monthly samplings, resp.) while these proportions were much lower for the other shedding patterns. In addition, around 50% of persistently highly seropositive cows were found to be persistent shedders, while seropositive cows with other patterns were mainly either non- or sporadic shedders.

#### 5.2.3. Immunofluorescence Assay (IFA)

In human medicine, IFA is commonly considered the reference diagnostic test and is the most frequently used worldwide [102]. It is accurate, highly sensitive, and specific [192]. The study by Rousset et al. [257] on goats demonstrated an overall good agreement between IFA and ELISA results. The study also reported that IFA results obtained on sera of aborting goats and of nonaborting goats were significantly different and were associated with occurrence of abortion [257]. Moreover, IFA is capable of detecting the two antigenic variants of *Coxiella burnetii* (phase I and phase II). As mentioned previously, in acute infections, anti-phase II antibodies are detected. In chronic infections, anti-phase I and anti-phase II antibodies are present but anti-phase I antibodies predominate [192]. As anti-phase II antibodies are present in all stages of infection, screening for epidemiological purposes is based on the detection of anti-phase II antibodies [257]. Cross-reactions with *Legionella micdadei* and *Bartonella* have been reported but do not cause any problem for the interpretation of quantitative results [259, 260]. In human medicine, IFA distinguishes IgG and IgM. Titers of anti-phase II IgG superior or equal to 200 and of anti-phase II IgM superior or equal to 50 correspond to an acute infection with a predictive value of 100%. An isolated high titer of IgM (≥50) can correspond to the beginning of an acute infection as long as the possibility of a false positive can be rejected. A chronic infection is characterized by a high titer in anti-phase I IgG (≥800) with a predictive value of 98% for a titer of 800 and a predictive value of 100% for a titer of 1600 [261]. The preferential use of IFA instead of CFT in veterinary medicine would be advantageous for diagnosis and control of Q fever at animal level [102]. IFA is currently the gold standard test for serology.

#### 5.2.4. Skin Test

A skin test method was proposed to investigate the cellular response and to improve detection of infected cows at herd level [128]. The skin test consists in an intradermal injection of extremely diluted inactivated vaccine (Coxevac, *CEVA-Santé Animale*, Libourne, France). The diluted vaccine induces an antigenic reaction. If the animal has previously been infected by Q fever, a nodule of variable size will appear at the site of injection. This test could easily be applied by rural practitioners.

#### 5.2.5. Negative Aspects of Indirect Diagnostic Methods

The dependence of CFT, IFA, and ELISA on the presence of antibodies limits their diagnostic value. Indeed, specific antibodies are often absent during the first 2 to 3 weeks of infection, making early diagnosis by serology difficult, if not impossible [200]. In Q fever, phase II antibodies can be detected within two weeks of infection in most cases and within three weeks 90% are seropositive. AFSSA [102] reports that a definitive diagnosis of human cases by IFA can only be confirmed one month and a half after the initial clinical signs. Cellular immunity also necessitates time before becoming detectable but the use of skin testing at herd level prevents from it being a problem as different stages of infection are present simultaneously in the herd. None of these tests are capable of confirming an etiological diagnosis at an individual level. Moreover, in acute infections, rheumatic factor, anti-mitochondrial antibodies, anti-nuclear antibodies, anti-smooth muscle antibodies, anticardiolipin and lupus anticoagulant, and other autoantibodies often present a marked increase in their plasmatic levels and can interfere with diagnostic assays [262]. A high prevalence of IgM against Epstein-Barr virus, cytomegalovirus, parvovirus, *Bordetella pertussis,* and *Mycoplasma pneumonia* has also been detected. Vardi et al. [262] concluded that diagnosis should not rely on a unique diagnostic approach. The global clinical and epidemiological context must be taken into account as well as the limitations of diagnostic assays. Bacteriological analyses are necessary to confirm or infirm any suspicion of Q fever [257].

### 5.3. Diagnosis by Histopathology

In acute cases of hepatitis, the presence of doughnut granulomas can be visualized in histopathology hepatic specimens but they are not pathognomonic of Q fever. However, in chronic infections, granulomas are less organized but bacteria can be detected in large vacuoles [192]. As mentioned above, in bovids, placentitis was found to be significantly associated with *Coxiella burnetii* infection [119]. On microscopic examination, an increased number of mononuclear cells (macrophages, lymphocytes, and plasma cells) can be identified in the chorionic stroma. Increased stromal collagen is also frequently observed. Furthermore, placental necrosis is significantly associated with the presence of *Coxiella burnetii*. Chorionic epithelial cells and villus tips are the most frequently affected. Infected trophoblasts are distended and contain basophilic granular to foamy material. The cytoplasms of infected cells appear bright red with the Macc stain, and their nuclei are commonly eccentric. A modified Koster's acid-fast (MAF) staining of fresh placenta smears is considered a good screening test, but confirmation by immunohistochemical techniques remains necessary [119]. In endocarditis lesions, *Coxiella burnetii* is visible as a voluminous intracytoplasmic mass within infected mononuclear cells [263]. 

## 6. Control Methods and Vaccination

In the case of a Q fever outbreak, sanitary and prophylactic measures should be applied at herd and human level, in order to limit disease transmission. Human and animal infections must be diagnosed early and treated immediately to prevent the development of chronic infections and secondary complications. With Q fever being a zoonosis, prophylaxis at herd level is fundamental. 

### 6.1. Control Methods

In The Netherlands, spread of manure from infected herds is forbidden for at least 90 days after suspicion of infection [130]. The effectiveness of this measure must be evaluated and modified if necessary. Arricau-Bouvery et al. [264] performed decontamination of feces of experimentally infected goats with calcium Cyanamid. Based on the results of this study, adding 0.5% of calcium Cyanamid to contaminated dung is an effective disinfectant. This measure, or its modified equivalent, should be applied worldwide if its effectiveness is proven. Transport of animals in and from infected areas should be strictly controlled and restricted to days without wind. 

In France, when Q fever has been diagnosed in a herd on a cheese-producing farm, milk of the aborted females must be discarded. Indeed, sale, transformation, and treatment of this milk are strictly forbidden (AFSSA, 2007). Milk of the remainder of the flock can be used for transformation, unless it is highly suspected that dairy products originating from these animals are dangerous for human consumption. In the latter case, the milk must be pasteurized at 72°C during 15 minutes or by an equivalent thermal treatment [265] (AFSSA, 2007). If Q fever is diagnosed on a farm producing raw milk for direct human consumption, sale of milk is forbidden during one year after the initial diagnosis of Q fever in an animal (AFSSA, 2007). 

In the UK, Health Protection Agency guidelines suggest the use of 2% formaldehyde, 1% Lysol, 5% hydrogen peroxide, 70% ethanol, or 5% chloroform for decontamination of surfaces, and spills of contaminated material should be dealt with immediately using hypochlorite, 5% peroxide, or phenol-based solutions (Health Protection Agency, 2010 at http://www.hpa.org.uk/deliberate_accidental_releases/biological). However, the guidelines note than decontaminating a large surface area is impossible. Moreover, Scott and Williams [266] found that formaldehyde vapour was ineffective in the absence of a high relative humidity. High-risk material (contaminated bedding, placenta, and aborted fetuses) should be buried with lime or incinerated. Treatment of manure with lime or calcium cyanide is also recommended before spreading, and spreading must be performed on a calm day.

At human level, prevention of exposure to animals or wearing gloves and masks during manipulation of animals or their litter is advised [102, 205]. Postexposure prophylaxis guidelines for the general population have been established in the USA [267, 268]. Doxycycline at a dose of 100 mg a day or 500 mg of tetracycline twice daily started 8–12 days after exposure is advised. No recommendation is available for pregnant women although cotrimoxazole has been suggested [267, 268].

Identification of an antibiotic capable of severely diminishing or completely stopping shedding would radically modify the management of *Coxiella* infection at a herd level. Currently, antibiotic treatment does not stop shedding [269, 270]. It could also prove useful to prevent infection of pregnant women by inhibiting shedding of bacteria by domestic cats and dogs. 

Pasteurization of all milk products should be performed if ingestion is proven an effective route of infection and after determination of the minimal infecting dose of bacteria. Awaiting further scientific research, caution is recommended especially for individuals at high risk of chronic infection.

### 6.2. Vaccination

Rodokalis et al. [97] suggested a followup of bovine and caprine herds by BTM analysis. In herds presenting a PCR-positive BTM result, pools of 10 individual milk samples should be tested by PCR to identify the shedding animals. If shedders are not very numerous, they can be eliminated and the other animals should be vaccinated. Rodokalis et al. [97] also suggested vaccination of herds in the proximity of infected herds or flocks. 

In animals, vaccines considered the most effective are composed of inactivated whole phase 1 bacteria [49]. Indeed, in goats, the inactivated phase I vaccine (Coxevac, CEVA-Santé Animale, Libourne, France) protects efficiently against abortion and has been shown to prevent bacterial shedding in vaginal mucus, feces, and particularly in milk. However, vaccination proved more effective in nulliparous animals than in parous animals. Furthermore, vaccination did not clear infection in previously infected goats [135, 236, 271] and cattle [272].

The study by Guatteo et al. [128, 272] on dairy cattle reported that the probability of becoming a shedder for vaccinated naïve nonpregnant bovids was 5-fold inferior to the probability for naïve bovids receiving a placebo. However, vaccination had no effect on the bacterial load shed. Vaccination of previously infected animals and of naïve animals during pregnancy proved ineffective. Guatteo et al. [128, 272] hypothesized that the immunodepression induced by pregnancy was responsible for the lack of effective immune response after vaccination. This explanation seems plausible, but a second study is necessary to reach confirmation of this hypothesis. No adverse effect was observed at the site of injection in this study. One abortion was reported in a vaccinated infected cow, but no further investigations were performed on this animal except for a PCR on vaginal mucus at abortion time that gave a negative result. 

The major problem associated with vaccination with inactivated phase 1 vaccine is the impossibility of distinguishing vaccinated and naturally infected animals using ELISA methods. At herd level, the effectiveness of a vaccination program could be evaluated by monitoring bacterial shedding in BTM. Currently, prophylaxis includes vaccination with the nonfully licensed inactivated phase I vaccine, Coxevac (CEVA-Santé Animale, Libourne, France), when a focus of Q fever is declared. This vaccine contains formalin-inactivated *Coxiella burnetii* (strain RSA 493/Nine Mile phase I) [273]. A recent study by Hermans et al. [274] reported the presence of bacterial DNA originating from the Coxevax vaccine in goat milk after inoculation. *Coxiella burnetii* DNA was detectable until 9 days after vaccination in quantities estimated by PCR up to approximately 100 genome equivalents per ml. The quantity of DNA detected in their study was around detection level. After the booster, the duration of vaccine-derived DNA excretion was shorter, quantitatively lower, and detectable in fewer animals than after the first inoculation. This finding induced the modification of the Dutch control strategy. Indeed, after the outbreak, testing BTM by PCR on dairy farms with at least 50 sheep or goats every two weeks and vaccination of all dairy goats and sheep with Coxevac became compulsory. After this study, a two-week interval between vaccination and BTM testing was imposed [274]. The study by De Cremoux et al. [139] demonstrated that vaccination in infected goat herds diminished clinical signs and overall vaginal shedding with vaccination being most effective in young susceptible animals. In addition, the duration of vaccine protection is not fully investigated. New vaccines, such as recombinant vaccines, have been developed [76]. Several of these vaccines have proven to be antigenic but nonprotective. Others still require an investigation of their effectiveness in field conditions [275]. An inactivated phase II vaccine, Chlamyvax FQ, has also been tested on animals but has failed to be effective [135]. 

Vaccination of humans against Q fever could be effective in certain areas. Several different vaccines have been developed since the discovery of *Coxiella burnetii*. In the Soviet Union in the 1960s, a live attenuated strain M-44 obtained after repeated passage through guinea pigs and mice was used extensively for vaccination. Because of the long-term persistence of the attenuated bacteria in animals and vaccinated human, this vaccine was never used in the West [276]. The Americans developed a chloroform-methanol residue vaccine based on the phase I Henzerling strain of *Coxiella burnetii* [277]. Despite being effective in protecting animals against aerosol challenges, humans were found to develop severe reactions to vaccination [277]. A trichloroacetic acid extract of phase I Nine Mile strain which comprised proteins and LPS was also used for vaccination. However, Kazar et al. [278] reported severe reactions in response to vaccination in humans. Vaccinating with phase I Nine Mile extracts treated with chloroform-methanol prevented severe reactions, but the loss of protective immunogenicity rendered these vaccines ineffective [279–282]. Currently, a formalin-killed whole-cell vaccine prepared from phase I Henzerling strain of *Coxiella burnetii* (Qvax, *CSL Limited*, Parkville, Victoria, Australia) is licensed in Australia [49]. This vaccine proved effective in several studies [283], but vaccination is only possible for individuals that have not previously been in contact with *Coxiella burnetii* to prevent serious reactions [17, 49, 284]. Screening is thus required before vaccination [285, 286], making this preventive measure time consuming and costly. Gefenaite et al. [287] performed a meta-analysis of previous studies on the effectiveness of Qvax vaccine. All the studies included in their analysis reported a protective effect of vaccination (average effectiveness after pooling raw data: 97%, CI: 94–99%). However, several biases were present in the latter studies. Gefenaite et al. [287] conclude that generalization of the results to the general population or to a specific risk group was not possible. The authors advised more blinded, randomized, and unbiased research on Qvax effectiveness. Development of recombinant protein subunit vaccines has proven disappointing [76, 288–290]. Before initiating a vaccination program, epidemiological knowledge of the area is necessary. Indeed, in endemic regions, vaccination is impossible for practical reasons (screening) and noneffective as a preventive measure. However, vaccination could be beneficial for at-risk individuals such as pregnant or immunocompromised individuals, farmers, veterinarians, abattoir workers, and research and reference laboratory personnel in contact with *Coxiella burnetii* [49]. Availability of vaccines causes a real problem in most countries and vaccination is currently not performed in Europe.

## 7. Perspectives for the Future

A better knowledge of Q fever would improve diagnostic procedures, control, and prevention of the disease in the future.

A better understanding of the bacterium and of its pathogenesis (entry into the cell, “sporulation-like” phenomenon, metabolism, mechanism of resistance to acidic conditions, infectivity of the bacterium, survival time in the environment under different management practices, etc.) is essential. The immune response of the infected host must also be investigated further. The risk factors for illness must be determined (infectious dose, potential danger linked to pets or not, etc.). Moreover, studies are required to define more precisely the incidence, clinical spectrum, treatment, morbidity, and mortality associated with Q fever in children. Further studies on risk factors could confirm or contradict results of previous studies, such as the studies by Whitney et al. [205] and McCaughey et al. [201]. Oral transmission of Q fever remains a controversial subject to this day. Further research is essential for the establishment of guidelines in case of an epidemic. Importantly, a high standard of laboratory diagnostic methods should be available in all accredited laboratories and a systematic search for *Coxiella burnetii* should be performed when clinical signs render the individual or animal suspected of Q fever. New guidelines for general practitioners and gynecologists should be established to increase the rapidity of diagnosis of clinical Q fever. Moreover, guidelines for prevention of infection of the medical staff and for prevention of transmission from one patient to another are necessary. The veterinary aspects of Q fever also necessitate further investigations. Indeed, the effect of antibiotic treatment on shedding of bacteria in ruminants has been insufficiently studied. Antibiotics have been suggested to diminish shedding by infected female animals but the effectiveness of this measure remains hypothetical. The prevalence and potential clinical consequences of Q fever in pets, wildlife, and other neglected domestic animals, such as pigs, must also be investigated. Veterinarians must include Q fever in their differential diagnoses of clinical cases whenever it cannot systematically be rejected as a potential diagnosis. Inactive whole-cell phase I vaccines have proved to be effective but present several limitations (e.g., effectiveness may not be observed in the short term, current impossibility of distinguishing vaccinated and naturally infected animals). Further research in this field and the development of new recombinant vaccines would permit a better management of foci of infection at a herd level but also in human populations in the future. Animal vaccination and vaccination of individuals at high risk of exposure or/and of severe clinical disease would diminish significantly the zoonotic risk. 

## 8. Conclusions

Q fever is an underestimated disease that remains poorly understood to this day. The lack of awareness of this disease leads to underdiagnosing and underreporting of Q fever cases. Q fever is ubiquitous, and the potential hosts for the infectious bacteria are extremely numerous. Q fever infections can have a significant economical impact on animal reproduction, animal trade, and the production and commercialization of animal products. In small ruminant flocks, the consequences of Q fever can be disastrous. Its zoonotic potential renders Q fever even more important. Human infections can lead to death. Furthermore, with the advances in human medicine, the number of immunosuppressed, premature, elderly, and chronically ill individuals has greatly increased compared to several years ago. Thus, the population predisposed to infection, and especially to chronic infection, has also greatly increased. Chronic infections can lead to severe consequences that necessitate intense medical treatment increasing public health costs and the patients' suffering. An early diagnosis and early initiation of treatment are essential to improve prognosis by preventing the development of a chronic infection or other potential complications associated with coxiellosis.

## Figures and Tables

**Table 1:** Relative epidemiological sensitivity and specificity (%) for different diagnostic methods.

Method evaluated	Method of reference	Species	Se	Sp	Reference
Agglutination	MI^1^	Cattle	94.3	95.5	[251]
ELISA	CF^2^	Human	99	88	[252]
ELISA	IFI^3^	Human	—	100	[253]
ELISA	Clinical signs	Human	94.8	—	[254]
IFI	Clinical signs	Human	90.6	—	[254]
CF	Clinical signs	Human	77.8	—	[254]
CF	IFI^3^	Human	—	90	[252]

^1^MI: microimmunofluorescence; ^2^CF: complement fixation; ^3^IFI: indirecte immunofluorescence.
